# Substrate Binding Protein DppA1 of ABC Transporter DppBCDF Increases Biofilm Formation in *Pseudomonas aeruginosa* by Inhibiting Pf5 Prophage Lysis

**DOI:** 10.3389/fmicb.2018.00030

**Published:** 2018-01-24

**Authors:** Yunho Lee, Sooyeon Song, Lili Sheng, Lei Zhu, Jun-Seob Kim, Thomas K. Wood

**Affiliations:** ^1^Department of Chemical Engineering, Pennsylvania State University, University Park, PA, United States; ^2^Department of Biochemistry and Molecular Biology, Pennsylvania State University, University Park, PA, United States

**Keywords:** PA4496, biofilm formation, swarming motility, bacteriophage Pf5, ABC transporter

## Abstract

Filamentous phage impact biofilm development, stress tolerance, virulence, biofilm dispersal, and colony variants. Previously, we identified 137 *Pseudomonas aeruginosa* PA14 mutants with more than threefold enhanced and 88 mutants with more than 10-fold reduced biofilm formation by screening 5850 transposon mutants (*PLoS Pathogens* **5**: e1000483, 2009). Here, we characterized the function of one of these 225 mutations, *dppA1* (PA14_58350), in regard to biofilm formation. DppA1 is a substrate-binding protein (SBP) involved in peptide utilization via the DppBCDF ABC transporter system. We show that compared to the wild-type strain, inactivating *dppA1* led to 68-fold less biofilm formation in a static model and abolished biofilm formation in flow cells. Moreover, the *dppA1* mutant had a delay in swarming and produced 20-fold less small-colony variants, and both biofilm formation and swarming were complemented by producing DppA1. A whole-transcriptome analysis showed that only 10 bacteriophage Pf5 genes were significantly induced in the biofilm cells of the *dppA1* mutant compared to the wild-type strain, and inactivation of *dppA1* resulted in a 600-fold increase in Pf5 excision and a million-fold increase in phage production. As expected, inactivating Pf5 genes PA0720 and PA0723 increased biofilm formation substantially. Inactivation of DppA1 also reduced growth (due to cell lysis). Hence, DppA1 increases biofilm formation by repressing Pf5 prophage.

## Introduction

*Pseudomonas aeruginosa* is a Gram-negative human pathogen that can be isolated from most environments, including soil, marshes, coastal marine habitats, plants, and mammal tissue (Bjarnsholt et al., 2010). It is an opportunistic human pathogen that causes infections of the pulmonary tract, urinary tract, and burn wounds (Balasubramanian and Mathee, 2009). Its ability to form biofilms is thought to contribute to the persistence of *P. aeruginosa* in lung infections (Ryder et al., 2007). Biofilms are a matrix-enclosed, surface-associated, multi-cellular assemblages that protect cells in hostile environments (Friedman and Kolter, 2004) such as phagocytosis (Hoiby et al., 2010). Biofilms are formed through the production of extracellular matrix composed of water, exopolysaccharides (EPS), nucleic acids, proteins, and lipids (Flemming and Wingender, 2010).

*Pseudomonas aeruginosa* is one of the most-studied microbes with respect to the genetic regulation taking place throughout the process of biofilm formation (Petrova and Sauer, 2009; Martínez-Gil et al., 2010); however, this regulation is not completely elucidated. For example, the genetic mechanism for matrix formation has not been fully elucidated (Franklin et al., 2011), and the complex role of the matrix is only beginning to be understood; for example, the interaction between the Pel polymer and extracellular DNA in *P. aeruginosa* (Jennings et al., 2015). The molecular mechanisms that regulate biofilm formation differ between strains, while some features are conserved in biofilm formation such as the formation of extracellular matrix (López et al., 2010). The formation and maintenance of *P. aeruginosa* PA14 (henceforth PA14) biofilm critically depends upon the production of two distinct EPS, alginate and Pel (Friedman and Kolter, 2004). Pel is a polymer of *N*-acetyl-β-D-glucosamine (GlcNAc) and *N*-acetyl-β-D-galactosamine (GalNAc) (Jennings et al., 2015), and *pel* mutations dramatically decrease biofilm formation as well as the pellicle that forms at the air-liquid interface (Friedman and Kolter, 2004). The regulation of the *pel* operon involves tyrosine phosphatase TpbA (Ueda and Wood, 2009) and the Las quorum sensing system (Sakuragi and Kolter, 2007).

In many Gram-negative bacteria, ATP binding cassette (ABC) transporters are used to transport substrates across the inner and outer membrane (Chung et al., 2009) using the energy derived from the hydrolysis of ATP (Moussatova et al., 2008). Transport systems including ABC transporters often recognize diverse substrates, such as ions, amino acids, and antibiotics (Moussatova et al., 2008), as well as materials required for biofilm formation (O’May et al., 2009; Iwai et al., 2010). In *P. aeruginosa*, ABC transport system DppBCDF is responsible for the uptake of dipeptides and tripeptides, which are used as nitrogen sources. The specificity of DppBCDF is controlled by its 5 substrate-binding proteins (SBPs) DppA1-A5 (PA14_58350, PA14_58360, PA14_58390, PA14_58420, and PA14_70200 in *P. aeruginosa* PA14, respectively), which function in the periplasmic space (Pletzer et al., 2014). ABC transporters function in DNA replication, protein degradation, membrane fusion, antibiotic efflux, signal transduction, and chemotaxis (Mai-Prochnow et al., 2015).

Filamentous bacteriophage such as f1, fd, and M13 of *Escherichia coli* are lysogens that, upon excision, are packaged as single-stranded phages that have a long filamentous shape; in *Pseudomonas* spp. they are designated as Pf phage (Pf1 to Pf6) (Mai-Prochnow et al., 2015). PA14 contains only Pf5 which is most closely related to Pf4 of *P. aeruginosa* PAO1 (63% homology) and somewhat related to Pf1 (Mooij et al., 2007). Specifically, Pf5 of PA14 is missing five genes of Pf4 of PAO1 (PA0715, PA0716, PA0728, and PA0729), and has three additional genes (PA14_49030, PA14_49010, and PA14_ 49020) (Mooij et al., 2007). Pf5 is thought not lead to phage particles (Mooij et al., 2007).

In *P. aeruginosa* biofilms, Pf1-related genes are highly expressed (up to 84-fold) suggesting that phage induction might be important for gene transfer within biofilms (Whiteley et al., 2001) and may be important as a stress response (Hui et al., 2014). Furthermore, there is a repeatable pattern of cell death and lysis in biofilms (Webb et al., 2003). Subsequently, Pf4 prophage were found to be essential for autolysis (programmed cell death) in *P. aeruginosa* PAO1 as deletion of the prophage prevented cells from undergoing cell death and from developing hollow regions in biofilms (Rice et al., 2009). Filamentous phage may also be secreted by the host without lysis (Hui et al., 2014). In *P. aeruginosa*, filamentous phage may also lead to the emergence of small-colony variants that might function in multicellular biofilm development (Webb et al., 2004). However, others have found that the prophage of PA14 and many clinical isolates do not affect small-colony variants which arise in biofilms (Mooij et al., 2007). In addition, Pf4 prophage facilitate biofilm formation by promoting the formation of liquid crystals of phage and *P. aeruginosa* PAO1 matrix polymers (Secor et al., 2015). Hence, filamentous bacteriophage are important for *P. aeruginosa* biofilm formation and may be involved in the production of small-colony variants.

Our goal here was to characterize the manner in which the SBP DppA1 (PA14_58350 in PA14 and PA4496 in *P. aeruginosa* PAO1), promotes biofilm formation in *P. aeruginosa;* this protein was identified via screen of 5850 transposon mutants that affected biofilm formation (Ueda and Wood, 2009). We found that inactivation of *dppA1* abolished biofilm formation in flow devices and delayed swarming. Using a whole-transcriptome analysis, we also determined that deletion of *dppA1* induced bacteriophage Pf5 genes during biofilm formation and led to a million-fold increase in lytic phage particles. Hence, DppA1 regulates Pf5 prophage to reduce biofilm formation.

## Materials and Methods

### Bacterial Strains, Plasmids, Primers, and Growth Conditions

Strains and plasmids used in this study are listed in **Table 1**. PA14 and its isogenic mutants were obtained from the Harvard Medical School (Liberati et al., 2006). Transposon insertion of the *dppA1* mutant was verified using a two-round PCR protocol^1^. Briefly, a PCR product was amplified from chromosomal DNA using primer pair GB 3a and Arb1D (**Table 2**); the product of this reaction was used as a template for the second reaction with primer pair GB 2a and Arb2A which was sequenced with two primers GB 2a and GB 4a (**Table 2**). The transposon insertions in *dppA2, dppA3, dppA4, dppA5*, and *dppC* were verified using flanking PCR with primer pairs Gb 4a/dppA2 R, Gb 4a/dppA3 R, dppA4 F/dppA4 R, Gb 4a/dppA5 R, and Gb 4a/dppC R, **Table 2**). *P. aeruginosa* and *E. coli* were routinely grown in Luria–Bertani (LB) medium at 37°C with aeration (250 rpm). Gentamicin (15 μg/mL) was used for growth of the *P. aeruginosa* transposon mutants, and carbenicillin (300 μg/mL) was used to maintain pMQ70 vector (Shanks et al., 2006). The specific growth rate was calculated using turbidity at 600 nm from 0.05 to 0.7.

**Table 1:** Strains and plasmids used in this study.

Strain and plasmids	Genotype or description	Reference
***P. aeruginosa***		
PA14	Wild-type strain	Liberati et al., 2006
PA14_58350 (PA4496, *dppA1*)	PA14_58350 Ω *Mar2xT7*, Gm^R^	Liberati et al., 2006
PA14_58360 (PA4497, *dppA2*)	PA14_58360 Ω *Mar2xT7*, Gm^R^	Liberati et al., 2006
PA14_58390 (PA4500, *dppA3*)	PA14_58390 Ω *Mar2xT7*, Gm^R^	Liberati et al., 2006
PA14_58420 (PA4502, *dppA4*)	PA14_58420 Ω *Mar2xT7*, Gm^R^	Liberati et al., 2006
PA14_70200 (PA5317, *dppA5*)	PA14_70200 Ω *Mar2xT7*, Gm^R^	Liberati et al., 2006
PA14_58450 (PA4504, *dppC*)	PA14_58450 Ω *Mar2xT7*, Gm^R^	Liberati et al., 2006
PA14_13660 (PA3885, *tpbA*)	PA14_13660 Ω *Mar2xT7*, Gm^R^	Liberati et al., 2006
PA14_24480 (PA3064, *pelA*)	PA14_24480 Ω *Mar2xT7*, Gm^R^	Liberati et al., 2006
PA14_19120 (PA3477, *rhlR*)	PA14_19120 Ω *Mar2xT7*, Gm^R^	Liberati et al., 2006
PA14_33700 (PA2396, *pvdF*)	PA14_33700 Ω *Mar2xT7*, Gm^R^	Liberati et al., 2006
PA14_49000 (PA0717)	PA14_49000 Ω *Mar2xT7*, Gm^R^	Liberati et al., 2006
PA14_48990 (PA0718)	PA14_48990 Ω *Mar2xT7*, Gm^R^	Liberati et al., 2006
PA14_48970 (PA0720)	PA14_48970 Ω *Mar2xT7*, Gm^R^	Liberati et al., 2006
PA14_48940 (PA0723)	PA14_48940 Ω *Mar2xT7*, Gm^R^	Liberati et al., 2006
PA14_48920 (PA0725)	PA14_48920 Ω *Mar2xT7*, Gm^R^	Liberati et al., 2006
PA14_48890 (PA0727)	PA14_48890 Ω *Mar2xT7*, Gm^R^	Liberati et al., 2006
***E. coli***		
HB101/pRK2013	*pro leu thi lacY Str^R^ endol^-^ recA^-^ r^-^ m^-^* with pRK2013, Km^R^	Ueda and Wood, 2009
TG1	*K12, lac–pro supE thi hsdD5 (F’ traD36 proA^+^B^+^ lacI^q^ lacZ M15*	Ueda and Wood, 2009
**Plasmids**		
pMQ70	Car^R^, Ap^R^, P_BAD_, expression vector	Shanks et al., 2006
pMQ-*dppA1*	Car^R^, Ap^R^, P_BAD_:: *dppA1*, complementation vector	This study

*Gm^*R*^ and Car^*R*^ indicate gentamicin, and carbenicillin resistance, respectively.*

**Table 2:** Primers used for DNA sequencing, complementation, phage excision, and quantitative real-time, reverse-transcription PCR (qRT-PCR) in this study.

Primer name	Primer sequence (listed 5′–3′)
**DNA sequencing**	
GB 2a	TGTCAACTGGGTTCGTGCCTTCATCCG
GB 3a	TACAGTTTACGAACCGAACAGGC
GB 4a	GACCGAGATAGGGTTGAGTG
Arb1D	GGCCAGGCCTGCAGATGATGNNNNNNNNNNGTAT
Arb2A	GGCCAGGCCTGCAGATGATG
dppA2 R	ATCTGCTCGTGGAAGATTCG
dppA3 R	GTAGCGGCTCAGGATGAAAG
dppA4 F	AACCTCGCTCTGGACAAGAA
dppA4 R	AACGGATTGATCACGTAGCC
dppA5 R	CAGATGTTCAGCTCGGGAAG
dppC R	CATCAGGTTGATCGCCAGTA
**Complementaion**	
DppA1-OE1	GCCCCCGCTAGCAAGAAGGAGATATACCATGCGTAGAAACGCCGTCATCCGC
DppA1-OE2	GCCCCCAAGCTTCTAGTGGTGGTGGTGGTGGTGCTTGCCGACGCTGACCTCGTAGAACG
**Page excision**	
Pf5-F	CAGGTAGAGCAGGATGTCG
Pf5-R	GCGTTGAACAGGAGGAAATGG
Pf5RF-F	AACAGTGAATTGCGGACAAGG
Pf5RF-R	ACGGTGGAAACATCCTGGC
Pf5-q-F2	GGTGCTCTGGAATCCGGGTGTTC
Pf5-q-R2	GTGGTCGAGGTCGGGAGTCATGG
rplU-F	AGGTTACCGCTGAAGTGGTTT
rplU-R	CCGGTGATCTTGATTTCAGTG
**qRT-PCR**	
PA0718-RT1	AGTCCCTACTACCTGCACCAAAC
PA0718-RT2	CGTACACAACGTGCCAGTACTTC
PA0722-RT1	CAACAGGCCTACCTGATTCC
PA0722-RT2	GGCTTTACGAAGAAGTGACG

### Complementation of *dppA1*

To complement the *dppA1* mutation, DppA1 was produced using the pBAD promoter in pMQ70 (Shanks et al., 2006). *dppA1* was amplified using Pfu DNA polymerase with primers DppA1-OE1 and DppA1-OE2 (**Table 2**). The PCR product was cloned into the *Nhe*I and *Hind*III cloning sites of pMQ70, creating pMQ-*dppA1*. The plasmid was confirmed by sequencing and transformed by conjugation (Ueda and Wood, 2009) using helper strain HB101/pRK2013.

### Biofilm Assay

Biofilm formation was examined in quiescent cultures using 96-well polystyrene plates and crystal violet staining (Ueda and Wood, 2009). Overnight-grown cultures of *P. aeruginosa* were diluted to a turbidity of 0.05 at 600 nm with fresh LB medium, then 300 μL of diluted bacterial culture were incubated in 96-well polystyrene plates for 4, 8, and 24 h. Six wells were used for each strain, and two independent cultures were used for each experiment. The *tpbA* and *pelA* mutants were used as a positive control and a negative control, respectively (Ueda and Wood, 2009).

Biofilm formation in flow cells was examined by diluting overnight cultures to a turbidity of 0.05 in 5% LB medium and pumping this culture through the flow-cell (BST model FC81, Biosurface Technologies, Bozeman, MT, United States) at 10 mL/h for 2 h (Ueda and Wood, 2010). Fresh 5% LB medium was added for 72 h, then the biofilms were stained with SYTO9 dye (5 μL/mL) for 20 min and were visualized using a TCS SP5 scanning confocal laser microscope (Leica Microsystems, Wetzlar, Germany). Nine random positions were chosen, and 25 images were taken for each position. Simulated three-dimensional images were obtained using IMARIS software (BITplane, Zurich, Switzerland), and the biomass, surface coverage, mean thickness, and roughness coefficient were determined using COMSTAT image-processing software (Heydorn et al., 2000).

### Small-Colony Variants

Small-colony variants were examined as described previously (Mooij et al., 2007). Approximately 2.5 × 10^8^ bacteria from overnight cultures were inoculated into glass tubes containing 5 mL of fresh LB medium and were incubated under static conditions for 2 to 5 days. Cells were removed from the tubes vortexed vigorously followed by gentle sonication (3W, 15 s) of 1 mL samples to dissolve bacterial aggregates completely. The sonicated cultures were diluted with PBS and plated on Congo-Red agar plates, which were incubated for 24 h. Two independent cultures were used for each strain along with three replicates for each culture.

### Prophage Excision

Excision was detected by PCR and by qPCR. Total DNA was extracted using the Ultra Clean Microbial DNA Isolation Kit (MO BIO Laboratories, Inc., Carlsbad, CA, United States). For the PCR method, excision was detected by amplifying the PA14 genome using primers PF5-F and PF5-R (these primers only give a band when the prophage has excised) (**Table 2**) and by amplifying circular prophage using primers PF5RF-F and PF5RF-R (these primers only give a band when the prophage has circularized) (**Table 2** and Supplementary Figure S1). For the qPCR method, total DNA (80 ng) was used with the PF5-q-F2 and PF5-q-R2 primers (**Table 2**) and the ABI Power SYBR^®^ Green RNA-to-CT^TM^ 1-Step Kit (Applied Biosystems, Carlsbad, CA, United States); these results were confirmed by using the Step OnePlus^TM^ Real-Time PCR System (Applied Biosystems, Foster City, CA, United States). Two independent cultures were used for each strain along with four replicates for each culture.

### Plaque Assay

The plaques were quantified by using a modified top-layer agar approach (Eisenstark, 1967). The bacterial lawns of the wild-type PA14 were prepared using early exponential-phase cells (10^8^ cells/mL) in LB media. For the phage stock solution, PA14 and the *dppA1* mutant were washed with fresh LB media (to remove any phage), inoculated (1%) into 25 mL of fresh LB media, and incubated with shaking for 8 h. Supernatants from the PA14 and the *dppA1* mutant were harvested by centrifugation (7,000 rpm for 15 min), diluted from 1/10^0^ to 1/10^4^, and mixed with 0.1 mL of wild-type bacteria (10^8^ cells/mL) in 4 mL of soft agar. The soft agar was poured onto the agar surface of a warm base plate. Plaques were observed after incubating for 6, 12, and 24 h. This plaque assay was performed with six independent cultures for each strain with three replicates for each culture.

### Live/Dead Assay

The percentage of live cells were determined using the LIVE/DEAD BacLight Bacterial Viability Kits L7012 (Molecular Probes, Inc., Eugene, OR, United States). The samples were harvested at the same turbidity at 600 nm after 3 to 5 h. The fluorescence emission spectrum (excitation 470 nm, emission 490 to 700 nm) of each cell suspension was measured using an Infinite M200 Pro plate reader (Tecan, Switzerland). The ratio was calculated of the integrated intensity of the portion of each spectrum from 510 to 540 nm (green, live cells) to that from 620 to 650 nm (red, dead cells) for each bacterial suspension. Isopropanol-treated cells (30 min) were used as the dead cell negative control.

### Motility Assay

Swarming motility was examined with BM-2 plates (62 mM potassium phosphate, 2 mM MgSO4, 10 mM FeSO4, 0.1% Casamino acids, 0.4% glucose, and 0.5% Bacto agar) (Overhage et al., 2008) after growth for 8 and 16 h. Three plates were tested for each culture, and two independent cultures were used. The *rhlR* mutant was used as a negative control (Köhler et al., 2000).

### EPS Assay

Exopolysaccharides production was quantified via Congo red staining (Ma and Wood, 2009). One mL of overnight-grown culture was washed with 1 mL of 1% tryptone (T-broth), resuspended in 1 mL T-broth, and sonicated three times at 3 W for 10 s. Bacterial suspensions in T-broth (500 μL) were incubated with 40 μg/mL Congo red with vigorous shaking (250 rpm) for 2 h. At that time, the absorbance of the supernatants was measured at 490 nm using a spectrophotometer. The *tpbA* and *pelA* mutants were used as positive control and negative controls, respectively (Ueda and Wood, 2009).

### Pyoverdine Assay

Production of pyoverdine was determined as described previously (Ren et al., 2005). Overnight cultures in LB broth were diluted to a turbidity of 0.05 at 600 nm in 25 mL of minimal succinate medium (6 g/L K_2_HPO_4_, 3 g/L KH_2_PO_4_, 1 g/L (NH_4_)_2_SO_4_, 0.2 g/L MgSO_4_⋅7H_2_O, and 9.15 g/L sodium succinate), and were incubated with aeration (250 rpm) for 4, 6, and 8 h. The absorbance at 405 nm of the supernatants was used for quantifying pyoverdine production which was normalized by cell turbidity.

### Pyocyanin Assay

Production of pyocyanin was quantified by the absorbance at 520 nm in acidic solution (Essar et al., 1990) and was normalized by cell turbidity. A 800 μL sample of culture grown in LB broth for 24 h was extracted with 480 μL of chloroform and then re-extracted into 800 μL of 0.2 N HCl to give a pink to deep red solution.

### Whole-Transcriptome Analysis

The *P. aeruginosa* genome array (Affymetrix, P/N 510596) was used to investigate differential gene expression in biofilm cells between wild-type PA14 and the *dppA1* mutant. After incubating in 250 mL LB medium with 10 g of glass wool for 7 h with aeration (250 rpm), biofilm cells were harvested from the glass wool, and the cell pellets were resuspended in RNA*later* (Ambion Inc., Austin, TX). Total RNA was isolated using the RNeasy Mini Kit (Qiagen Inc., Valencia, CA, United States) (Ren et al., 2004a) and a bead beater (Biospec, Bartlesville, OK, United States). cDNA synthesis, fragmentation, and hybridizations were performed as described previously (González Barrios et al., 2006). If the gene with the larger transcription rate did not have a consistent transcription rate based on the 13 probe pairs (*p*-value less than 0.05), these genes were discarded. A gene was considered differentially expressed when the *p*-value for comparing two chips was lower than 0.05 (to assure that the change in gene expression was statistically significant and that false positives arise less than 5%) and if their fold change is higher than standard deviation for the whole genome (Ren et al., 2004b) (1.8-fold). The expression data have been submitted to the NCBI Gene Expression Omnibus (GSE24638). The STRING online database was used to construct a protein-protein interaction network among the proteins (Szklarczyk et al., 2015).

### Quantitative Real Time, Reverse Transcription-PCR (qRT-PCR)

Quantitative real time, reverse transcription-PCR was performed using the StepOnePlus^TM^ Real-Time PCR System (Applied Biosystems, Foster City, CA, United States). Expression levels were measured using total RNA isolated from biofilm cells under the same conditions as the microarray experiment (independent samples were used). The primers for qRT-PCR are listed in **Table 2**. The values were normalized using a housekeeping gene *rplU* (Kuchma et al., 2007).

## Results

### DppA1 Increases Biofilm Formation

**FIGURE 1:**
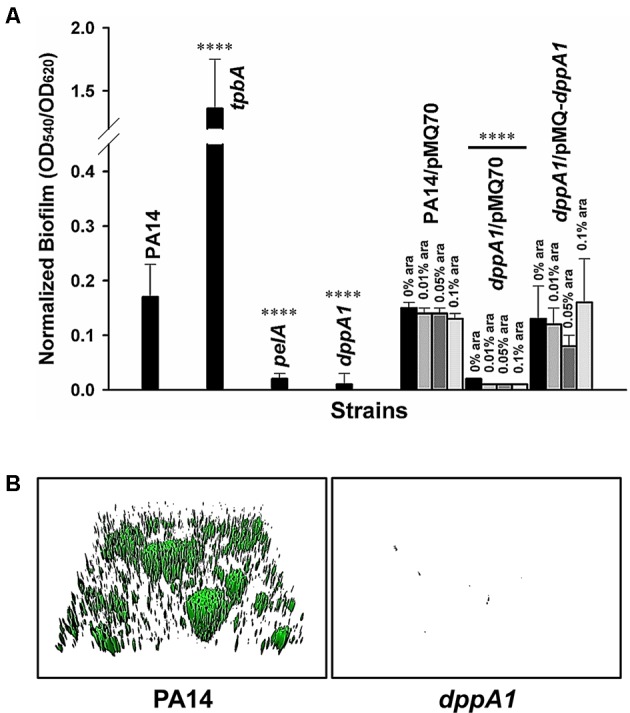
*dppA1* decreases biofilm formation. **(A)** Total biofilm formation (at the liquid/solid and air/liquid interfaces) in polystyrene plates at 37°C in LB after 24 h. Six wells were used for each independent culture, and the averages of two independent experiments ±SD are shown. **(B)** Biofilm formation in flow cells with 5% LB after 72 h. Images were produced by IMARIS, and representative images are shown. Asterisks above the columns represent significance based on the unpaired Wilcoxon *t*-test (^∗∗∗∗^*p* < 0.0001).

Previously, by screening 5850 transposon mutants for altered biofilm formation, we identified 137 transposon mutants with over threefold enhanced biofilm formation and 88 transposon mutants with 10-fold reduced biofilm formation (Ueda et al., 2009). Among these, inactivating *dppA1* decreased biofilm formation by 68-fold after 24 h in LB medium in quiescent 96-well plates (**Figure 1A**). In comparison, the isogenic positive control, *tpbA*, increased biofilm eightfold, and the negative control, *pelA*, decreased biofilm ninefold. The decreased biofilm formation by inactivating *dppA1* was restored by complementing with plasmid PMQ-*dppA1* (**Figure 1A**). Hence, DppA1 increases biofilm formation in PA14.

To verify these static biofilm formation results from 96-well plates, we investigated biofilm formation in flow cells. Critically, the *dppA1* mutant had no biofilm formation in the flow-cell under these conditions while the wild-type PA14 strain formed a robust biofilm (**Figure 1B**). These results were quantified using COMSTAT statistical analysis which showed 0% biomass, 0% mean thickness, and 0.14% surface coverage at 72 h for the *dppA1* mutant (**Table 3**). These results confirmed that DppA1 positively influences biofilm formation in both static and flow conditions.

**Table 3:** COMSTAT analysis for biofilms of PA14 and the *dppA1* mutant in flow cells.

COMSTAT values	PA14	*dppA1*
Biomass (μm^3^/μm^2^)	7 ± 4	0 ± 0
Surface coverage (%)	7 ± 4	0.14 ± 0.02
Mean thickness (μm)	14 ± 9	0 ± 0
Roughness coefficient	1.4 ± 0.2	2 ± 0

*Biofilms were evaluated after 72 h of development in 5% LB medium at 37°C.*

We also investigated whether the effect on biofilm formation was specific for DppA1 since there are four other substrate binding proteins for dipeptides associated with ABC transporter DppBCDF. We found that the mutations in the other substrate binding protein *dppA2, dppA3, dppA4*, and *dppA5* have no effect on biofilm formation, i.e., these mutants have the same biofilm level as the wild-type strain. Furthermore, we found that there was no effect on biofilm formation upon mutating the permease DppC. Therefore, the dramatic biofilm reduction phenotype is specific for inactivating DppA1.

### Small-Colony Variants

Since DppA1 increases biofilm formation dramatically, we also investigated whether DppA1 increases the formation of small-colony variants. Inactivating *dppA1* reduces the production of small-colony variants by 20-fold (40% for wild-type vs. 2% for the *dppA1* mutant, images of colonies not shown). Production of DppA1 from pMQ-*dppA1* increased the number of small-colony variants to about half that of the wild-type strain; hence, the large decrease in small-colony variants could be partially complemented.

### DppA1 Increases Swarming

**FIGURE 2:**
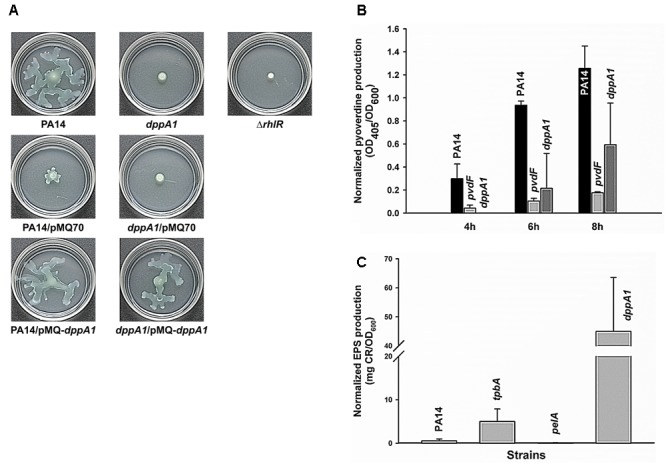
*dppA1* decreases swarming and pyoverdine production and decreases exopolysaccharides (EPS). **(A)** Swarming motility at 37°C after 15 h. **(B)** Pyoverdine production after 4, 6, and 8 h. **(C)** EPS production of PA14 strains after 24 h at 37°C. The averages of two independent experiments ±SD are shown.

Since *dppA1* encodes a putative binding protein component of an ABC transporter, and many transport genes control swarming motility in *P. aeruginosa* (e.g., PA14_48280, PA14_40620, PA14_30660, PA14_20110, and PA14_16890) (Yeung et al., 2009), we examined swarming motility for *dppA1* and found that this mutation abolished swarming in the first 8 h; this phenotype could be complemented by expressing *dppA1* in trans (**Figure 2A**). An *rhlR* mutant was used a negative control for swarming (Köhler et al., 2000). Critically, swarming resumed for the *dppA1* mutant after an additional 8 h. These results suggest a small percentage of cells recovered after some event to give the wild-type swarming phenotype.

### DppA1 Decreases EPS Production

Given the large reduction in biofilm formation upon inactivating DppA1, the biofilm-associated factors in PA14, including EPS, pyocyanin, and pyoverdine were assessed in the *dppA1* mutant. Since Congo red (CR) binds extracellular matrix components including EPS in many bacteria (Friedman and Kolter, 2004), EPS was assayed through Congo red staining with the PA14 and the *dppA1* mutant. Surprisingly, the *dppA1* mutant produced 85-fold more EPS than wild-type PA14 (**Figure 2C**). For these EPS experiments, the *pelA* mutant was used as a negative control, and the *tpbA* mutant was used as a positive control (Ueda and Wood, 2009).

Moreover, since pyocyanin is a redox-active pigment secreted by *P. aeruginosa* that affects the structure of biofilms (Dietrich et al., 2008), and iron serves as a signal in *P. aeruginosa* biofilm development through pyoverdine iron acquisition (Banin et al., 2005), we investigated whether pyocyanin and pyoverdine production is altered by in the *dppA1* mutant. The *phzM/phzS* (Mavrodi et al., 2001) and *pvdF* mutants (Banin et al., 2005) were used as negative controls for pyocyanin and pyoverdine production, respectively. We found there was no difference in pyocyanin production between PA14 and the *dppA1* mutant LB medium at 10 h and 24 h (data not shown), while the *dppA1* mutant decreased pyoverdine production twofold compared to PA14 at 8 h (**Figure 2B**).

### DppA1 Represses Bacteriophage Pf5 in Biofilm Cells

To determine how the *dppA1* mutation affects biofilm formation, a whole-transcriptome analysis was performed to compare gene expression in early biofilm formation (7 h) for the *dppA1* mutant relative to the wild-type (grown on glass wool). Surprisingly, since *dppA1* is not near the Pf5 locus, the whole-transcriptome data showed that inactivation of *dppA1* significantly induced 10 bacteriophage Pf5 genes (**Table 4**). To confirm the induction of the prophage Pf5 genes, expression of PA0718 and PA0722 were determined by qRT-PCR. Using total RNA isolated from biofilm cells of PA14 and *dppA1*, we found that PA0718 and PA0722 were induced 30 ± 4-fold and 41 ± 4-fold in the *dppA1* mutant compared to PA14, respectively.

**Table 4:** Summary of the differentially expressed biofilm genes related to bacteriophage Pf5 for the *dppA1* mutant vs. wild-type PA14 in LB medium for 7 h at 37°C.

PAO1 ID	PA14 ID	Gene name	Fold change	Description
PA0717	PA14_49000		7.0	Hypothetical protein
PA0718	PA14_48990		12.1	Hypothetical protein
PA0719	PA14_48980		7.5	Hypothetical protein
PA0720	PA14_48970		6.5	Single-stranded binding protein
PA0721	PA14_48960		10.6	ABC transporter permease
PA0722	PA14_48950		9.2	Hypothetical protein
PA0723	PA14_48940	*coaB*	2.8	Coat protein B
PA0725	PA14_48920		5.7	Head virion protein G6P
PA0726	PA14_48910	*zot*	3.2	Zona occludens toxin
PA0727	PA14_48890		2.1	Hypothetical protein

*Raw data for the two DNA microarrays are available using GEO series accession number GSE24638.*

**FIGURE 3:**
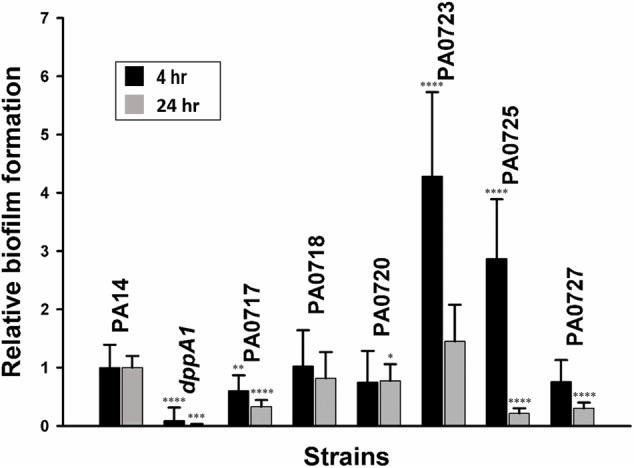
*dppA1* influences biofilm formation through bacteriophage Pf5. Relative biofilm formation in polystyrene plates at 37°C in LB after 4 and 8 h due to inactivating bacteriophage Pf5 genes. Six wells were used for each culture. The averages of two independent experiments ±SD are shown. Asterisks above the columns represent significance based on the unpaired Wilcoxon *t*-test relative to PA14 for each time (^∗∗∗∗^*p* < 0.0001, ^∗∗∗^*p* < 0.001, ^∗∗^*p* < 0.01, and ^∗^*p* < 0.05).

Based on these results; i.e., that inactivation of *dppA1* induced bacteriophage Pf5 and reduced biofilm formation, we hypothesized that deletion of individual bacteriophage Pf5 genes should increase biofilm formation. In agreement with this hypothesis, we found that the PA0723 and PA0725 mutants exhibited 4 ± 1- and 3 ± 1-fold increase in biofilm formation in LB medium at 4 h, respectively (**Figure 3**). Therefore, *dppA1* represses, either directly or indirectly, expression of Pf5 genes which results in increased biofilm.

### DppA1 Decreases Pf5 Excision

Since the *dppA1* deletion increases transcription of the Pf5 phage locus, we checked for prophage excision in this strain relative to the wild-type strain by quantifying the presence of re-circularized prophage via PCR for a pair of primers that only give a PCR band if the circularized prophage is formed (Supplementary Figure S1). As expected, we found via PCR that the *dppA1* deletion led to a 10-fold increase in excised, circularized prophage compared to the wild-type strain as well as a corresponding 10-fold increase in the chromosomal deletion area.

To corroborate and better quantify these PCR results, we performed qPCR with *rplU* as the housekeeping gene and found that the *dppA1* deletion had 600 ± 1-fold greater Pf5 phage excision from the chromosome compared to the wild-type strain (0.1% vs. 0.00017%) (Supplementary Table S1). Hence, DppA1 either directly or indirectly represses phage excision in the wild-type strain.

### DppA1 Decreases Plaque Formation, Increases Growth, and Reduces Cell Lysis

The induction of Pf5 phage genes, the increased Pf5 excision, the increase in EPS, and the delay in swarming motility, all suggest that inactivating DppA1 results in active cell lysis. Hence, we measured plaque formation with the supernatants of the *dppA1* mutant and wild-type and found an explosive 1.7 million-fold increase in plaque formation when DppA1 is inactivated (1.5 ± 0.3 × 10^8^ vs. 0.9 ± 0.5 × 10^2^ pfu/mL) after growth for 8 h in rich medium (turbidity around 3). Note that we used the wild-type strain as is routinely done (Hui et al., 2014) to enumerate the number of Pf5 phage that are capable of superinfection; these superinfective phage arise rapidly (Rice et al., 2009); hence, the large change in phage found is for the superinfecting form of Pf5.

**FIGURE 4:**
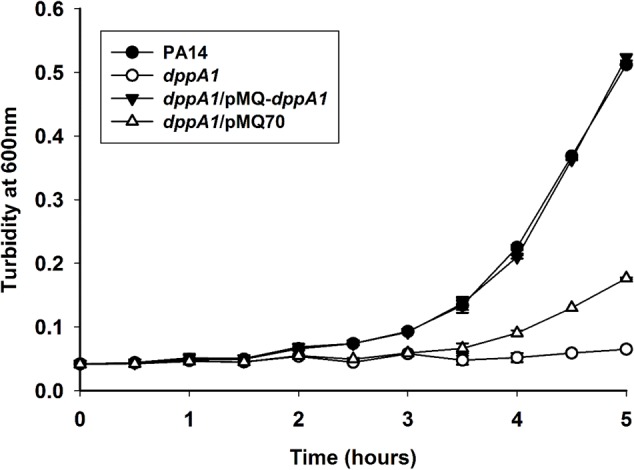
*dppA1* decreases growth. Cells were grown in LB broth at 37°C with shaking. The averages of two independent experiments ±SD are shown.

Corroborating the results showing extraordinary phage production, growth of the *dppA1* mutant is initially dramatically slower than the wild-type due to phage lysis (**Figure 4**). This slow-growth phenotype was completely complemented by producing DppA1 from plasmid pMQ-*dppA1.*

We reasoned that the increase in plaque production should lead to an increase in lysis. Confirming this, total protein increased twofold in the supernatants of the *dppA1* mutant vs. the wild-type strain. In addition, cell death was confirmed via Live/Dead staining where all of the wild-strain were alive after 5 h but 50% of the *dppA1* mutant were dead (isopropanol-treated cells were used as the dead cell negative control) (Supplementary Table S2).

### DppA1 Is a Substrate-Binding Protein

To provide insights into how DppA1 regulates biofilm formation and phage excision, we performed a protein domain analysis using the Conserved Domain Search Service^2^ and Basic Local Alignment Search Tool^3^ from NCBI, which showed that DppA1 (537 aa, PA14_58350 in *P. aeruginosa* PA14 and PA4496 in *P. aeruginosa* PAO1) contains a domain belonging to substrate-binding component of an ABC-type dipeptide import system PBP2 (PBP2_DppA_like, NCBI conserved domains accession no. cd08493). DppA1 has been shown to transport 72 of 246 tested dipeptides and 3 of 14 tripeptides tested (Pletzer et al., 2014). The PBP2 family members bind dipeptides and some tripeptides and are involved in chemotaxis toward dipeptides. The PBP2 superfamily also includes the ligand-binding domains from ionotropic glutamate receptors, LysR-type transcriptional regulators (Anantharaman et al., 2001), and unorthodox sensor proteins involved in signal transduction (Tam and Saier, 1993). Hence, there is precedent for SBPs such as DppA1 acting as regulators. However, DppA1does not contain any DNA/RNA binding domains.

To confirm the role of DppA1 as a dipeptide binding protein, we measured growth of the *dppA1* mutant on the dipeptides Phe-Pro and Gly-Glu as the sole carbon and energy sources. In contrast to the wildtype, the *dppA1* mutant cannot grow on these two dipeptides (Supplementary Figure S2). As a negative control, the wild-type could not grow on M9 buffer that lacks a carbon source, and as a positive control, the unrelated PA4499 mutation did not affect growth on dipeptides. Hence, DppA1 is a *bona fide* dipeptide binding protein involved in their transportation.

Since Pf5 in *P. aeruginosa* PA14 lacks a few proteins of Pf4 found in *P. aeruginosa* PAO1, we hypothesized that DppA1 may be related to PA0716, which is also a component of an ABC transporter of Pf4 in *P. aeruginosa* PAO1 (441 aa); PA0176 contains an ATP-binding cassette domain of nickel/oligopeptides specific transporters (ABC_NikE_OppD_transporters, NCBI conserved domains accession no. cd03257), and also seems to be a putative AbiEii toxin of type IV toxin/antitoxin systems (Dy et al., 2014). However, DppA1 and PA0716 belong to different ABC transporter families, and the amino acid identity between DppA1 and PA0716 is only 7%.

## Discussion

Many studies have shown that bacteriophage are important in biofilm development in both Gram-positive (Stanley et al., 2003) and Gram-negative bacteria (Whiteley et al., 2001; Domka et al., 2007), especially under conditions where the cells are stressed, such as those that exist in biofilms (Domka et al., 2007). Cell death and lysis occur in biofilms, and biofilm killing is related to filamentous phage (Webb et al., 2003). Furthermore, the emergence of small-colony variants and biofilm development is related to phage (Webb et al., 2004). Here we found that SBP DppA1 helps facilitate the growth on dipeptides and increases PA14 biofilm formation 68-fold. Critically, we determined that the mechanism by which DppA1 increases biofilm formation: DppA1 directly or indirectly represses Pf5 prophage genes which prevents cell lysis and a million-fold production of active phage.

Although the importance of Pf phage in *P. aeruginosa* biofilm formation is well-documented, such as the formation of extracellular liquid crystals by Pf4 with alginate and extracellular DNA (Secor et al., 2015) and the hyper-mutability of Pf4 during biofilm evolution (McElroy et al., 2014), our results shed light on how this phage is regulated, through the unexpected mechanism of a SBP DppA1. Clearly DppA1 helps to limit phage lysis and thereby increases biofilm production by reducing excess phage production; this appears to be at least part of the unknown mechanism by which Pf phage generate diversity in biofilms that was alluded to previously (Hui et al., 2014).

Furthermore, the mechanism we found here for DppA1 for controlling phage production and biofilm formation is distinct. For example, in contrast to our results where excessive phage production is deleterious for biofilm formation, previous work has shown that explosive cell lysis via an endolysin from cryptic prophage is beneficial for biofilm formation since it produces extracellular DNA that helps to initiate biofilm production (Turnbull et al., 2016). Therefore, phage production and phage-related genes must be regulated well to control biofilm formation.

Further work is clearly required to determine the mechanism by which DppA1 represses Pf5 prophage genes, either directly or indirectly, through dipeptide transport. However, it is tempting to speculate that the dipeptides transported by DppA1 in the periplasm are relayed as a signal to the Pf5 phage integrase/excisionase (PA14_48880) or some other phage regulator to limit phage lysis when nutrients are plentiful (extracellular proteins are readily degraded by the extracellular proteases of PA14). In contrast, when nutrients are depleted, the dipeptides transported by DppA1 are reduced, and Pf5 phage are produced to drive cell evolution and to create voids in biofilms to facilitate dispersal (inactivation of *dppA1* would mimic a no peptide condition). Such biofilm voids and dispersal have been shown to be dependent on the related phage, Pf4 (Rice et al., 2009), and biofilm dispersal has long been known to be governed by changes in nutrient levels (Sauer et al., 2004). Therefore, the dipeptide concentrations in the cells, governed by DppA1 transport, may serve as a simple indicator of external nutrient levels and control biofilm development.

## Author Contributions

TW conceived the project. YL, SS, LS, LZ, and J-SK conducted the experiments. YL, SS, and LS analyzed the data. YL, SS, and TW co-wrote the manuscript.

## Conflict of Interest Statement

The authors declare that the research was conducted in the absence of any commercial or financial relationships that could be construed as a potential conflict of interest.

## Footnotes

1 http://ausubellab.mgh.harvard.edu/cgi-bin/pa14/productionmethods.cgi

2 https://www.ncbi.nlm.nih.gov/Structure/cdd/wrpsb.cgi

3 https://blast.ncbi.nlm.nih.gov/Blast.cgi
