# Supplementary material for: Substrate Binding Protein DppA1 of ABC Transporter DppBCDF Increases Biofilm Formation in Pseudomonas aeruginosa by Inhibiting Pf5 Prophage Lysis
Source: Front Microbiol. 2018 Jan 24;9:30. doi: 10.3389/fmicb.2018.00030 (PMC5787571; doi:10.3389/fmicb.2018.00030)
Supplement: Supplementary file 1 [file Table_1.pdf]

SUPPLEMENTAL INFORMATION

Substrate Binding Protein DppA1 of ABC Transporter DppBCDF Increases Biofilm Formation in *Pseudomonas aeruginosa* by Inhibiting Pf5 Prophage Lysis

Yunho Lee[1Δ], Sooyeon Song[1Δ], Lili Sheng[1Δ], Lei Zhu[1], Jun-Seob Kim, and Thomas K. Wood[1,2]*

[1]Department of Chemical Engineering and [2]Department of Biochemistry and Molecular Biology, Pennsylvania State University, University Park, PA, 16802

*To whom correspondence should be addressed: tuw14@psu.edu

[Δ]These authors contributed equally.

Table S1. Detection of Pf5 excision by qPCR. Ct values for the excised genome region using primer PF5-q-F2 and PF5-q-R2 are shown. The reference gene was *rplU*. qPCR was performed using two independent cultures for each strain with four replicates for each culture.

**Strain**	***rplU* (Ct)**	**Pf5 (Ct)**	**ΔCt Ct(Pf5)-Ct(*rplU*)**	**Proportion of excised $2^{(-\Delta Ct)}$**
PA14	12.7 ± 0.2	25.24 ± 0.07	12.5	1.7E-4
dppA1	12.81 ± 0.06	16.11 ± 0.04	3.3	1.0E-1

Table S2. Live/dead assay of *dppA1* vs. *P. aeruginosa* wild-type. The fluorescence emission spectrum (excitation 470 nm, emission 490 to 700 nm) of each cell suspension was measured in a fluorescence spectrophotometer. The ratio was calculated for the integrated intensity of the portion of the spectrum between 510 to 540 nm (green) and that between 620 to 650 nm (red) for each bacterial suspension.

Standard curve data. The dead cells were prepared using 70% isopropanol-treated cells incubated for 30 min at 37 °C.

Ratio of Live:Dead cells	Green (510 to 540 nm)	Red (620 to 650 nm)	Green/red fluorescence ratio
0:100	204,858	2,561,075	0.08
10:90	322,853	260,415	1.24
50:50	584,700	159,738	3.66
90:10	1,415,333	95,183	14.87
100:0	1,606,453	72,975	22.01

Analysis of relative viability of PA14 and *dppA1* planktonic cells in a fluorescence microplate reader**.**

Strains	Green (510 to 540 nm)	Red (620 to 650 nm)	Green/red fluorescence ratio	Fold	Percentage
PA14 #1	7,546,475	28,823	26.18	–	–
PA14 #2	7,915,775	31,655	25.01	–	–
dppA1 #1	1,775,588	135,803	13.07	-2.00	-50.1%
dppA1 #2	2,102,683	165,630	12.70	-1.97	-49.2%

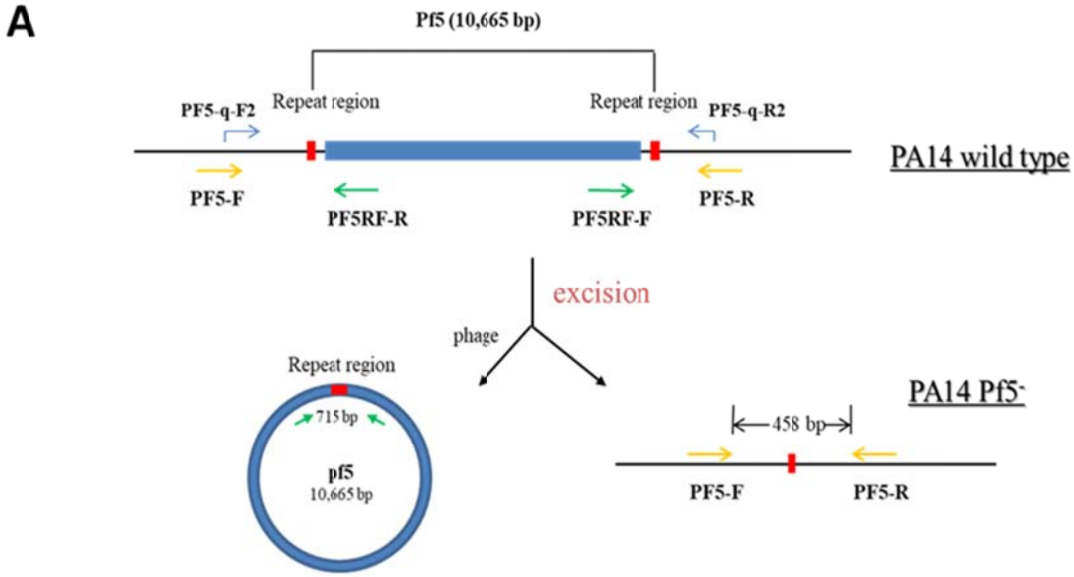

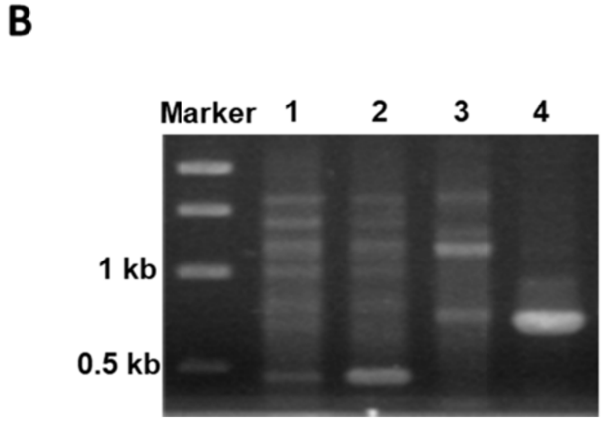

Figure S1. Detection of prophage excision. (**A**) Schematic of Pf5 excision. Pf5 is 10,665 bp. The three primer sets used in this study for Pf5 excision are indicated by arrows. PF5-F and PF5-R are only able to amplify the remnant left in the of PA14 genome after excision of Pf5; if Pf5 excises, the PCR product of Pf5-F and Pf5-R of the chromosome is 458 bp. Similarly, PF5-q-F2 and PF5-q-R2 primers were used for qPCR to determine if prophage excision occurred (**Table S1**). PF5RF-F and PF5RF-R primers will only be able to amplify the circularized prophage; if Pf5 circularizes, the PCR product should be 715 bp. (**B**) Agarose gel to quantify Pf5 excision by PCR. Lane 1: PF5-F and PF5-R primer PCR for the PA14 genome, Lane 2: PF5-F and PF5-R PCR for the *dppA1* genome, Lane 3: PF5RF-F and PF5RF-R PCR for circularized prophage in PA14, and Line 4: PF5RF-F and PF5RF-R PCR of circularized prophage in the *dppA1* mutant.

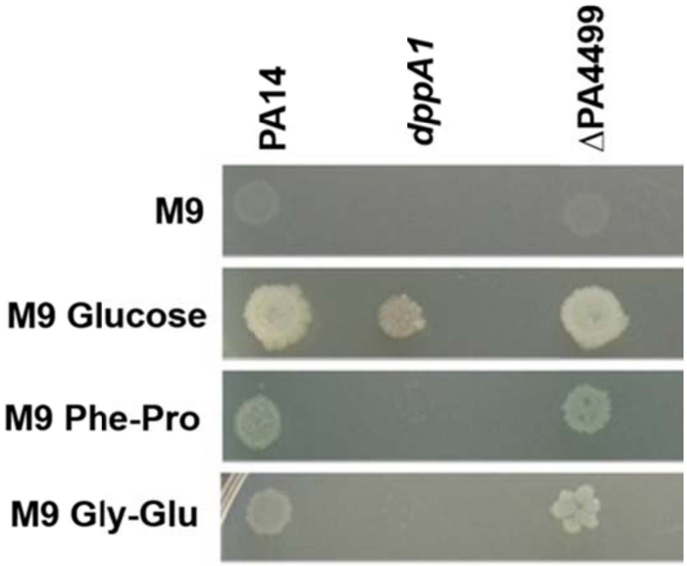

Figure S2. DppA1 is required for growth on dipeptides. Cells (1 μL at a turbidity of 0.05 at 600 nm) were diluted and plated on M9 minimal medium agar plates (M9 glucose, M9 Phe-Pro, and M9 Gly-Glu) and incubated for 3 days. ΔPA4499 was used as a positive control for growth (i.e., a transposon mutation in a non-related gene). Three independent cultures were used for each strain.
